# In Vivo Functional Assay in Fish Gills: Exploring Branchial Acid-Excreting Mechanisms in Zebrafish

**DOI:** 10.3390/ijms23084419

**Published:** 2022-04-16

**Authors:** Shang-Wu Shih, Jia-Jiun Yan, Yi-Ling Tsou, Shao-Wei Lu, Min-Chen Wang, Ming-Yi Chou, Pung-Pung Hwang

**Affiliations:** 1 Institute of Cellular and Organismic Biology, Academia Sinica, Taipei 11529, Taiwan; d08b21001@ntu.edu.tw (S.-W.S.); c1208940@gmail.com (J.-J.Y.); amydc10@gmail.com (Y.-L.T.); bluerclouder@gmail.com (S.-W.L.); mcwinlab@gmail.com (M.-C.W.); 2 Department of Life Science, National Taiwan University, Taipei 10617, Taiwan; mingyichou@ntu.edu.tw

**Keywords:** scanning ion-selective electrode technique, in vivo functional assay, H^+^ excretion, adult gills, zebrafish

## Abstract

Molecular and physiological analyses in ionoregulatory organs (e.g., adult gills and embryonic skin) are essential for studying fish ion regulation. Recent progress in the molecular physiology of fish ion regulation was mostly obtained in embryonic skin; however, studies of ion regulation in adult gills are still elusive and limited because there are no direct methods for in vivo functional assays in the gills. The present study applied the scanning ion-selective electrode technique (SIET) in adult gills to investigate branchial H^+^-excreting functions in vivo. We removed the opercula from zebrafish and then performed long-term acid acclimation experiments. The results of Western blot and immunofluorescence showed that the protein expression of H^+^-ATPase (HA) and the number of H^+^-ATPase-rich ionocytes were increased under acidic situations. The SIET results proved that the H^+^ excretion capacity is indeed enhanced in the gills acclimated to acidic water. In addition, both HA and Na^+^/H^+^ exchanger (Nhe) inhibitors suppressed the branchial H^+^ excretion capacity, suggesting that H^+^ is excreted in association with HA and Nhe in zebrafish gills. These results demonstrate that SIET is effective for in vivo detection in fish gills, representing a breakthrough approach for studying the molecular physiology of fish ion regulation.

## 1. Introduction

Body fluid acid–base homeostasis is a tightly controlled process in all biological systems in which acids are excreted or bicarbonate is absorbed/regenerated to maintain proper cellular activities and physiological processes. Adult gills and embryonic skin are the main ionoregulatory organs for internal osmotic and acid–base homeostasis in fish. These organs possess a large number of ionocytes expressing ion transporters that transport ions across the epithelium [1,2,3,4,5,6]. Recently, an evolutionary progression hypothesis was proposed for the acid excretion machinery in teleosts, including apical H^+^-ATPase (HA)-mediated and apical Na^+^/H^+^ exchanger (Nhe)-mediated acid excretion [7]. After invading hypotonic freshwater (FW) habitats, some teleosts (e.g., lamprey and zebrafish) evolved to develop apical HA-mediated H^+^ excretion machinery to actively excrete H^+^ against an environmental pH gradient [7,8,9], which may enable them to tolerate more acidic conditions. Indeed, zebrafish can be well-acclimated to a very low pH water environment (pH 4) [8,10,11,12], making this organism an ideal model for studying acid–base regulation and the underlying molecular mechanisms [13,14,15].

Different subtypes of zebrafish ionocytes in adult gills and embryonic skins have been identified in previous works, including H^+^-ATPase-rich (HR) ionocytes responsible for H^+^ excretion [8,9,15]. The scanning ion-selective electrode technique (SIET), a non-invasive electrophysiology method, was adopted to detect the real-time acid excretion function of HR ionocytes in intact zebrafish embryonic skin and demonstrate the elevated acid excretion in response to acidic environments [9,16,17]. However, direct information on the branchial H^+^-excreting function in zebrafish is still absent because of the difficulty to directly analyze the ion transport functions of the gills. Though some studies have developed methods to culture branchial cells or gill organs [18,19,20,21], functional analyses on these in vitro culture systems may not reflect the real physiological states of fish. So far, there are no real-time and direct methods for in vivo functional assays in adult gills. The main reason is that the gills are protected and covered by the opercula, which impede the operation and accessibility of the SIET or other electrophysiological techniques. As such, we do not really understand the physiological mechanisms in branchial ion transport, which is key for understanding body fluid ionic and acid–base homeostasis in adult fish.

To address this difficulty mentioned above, the present study removed the opercula and exposed the gills, allowing us to detect the ion activities in the gills with an ion-selective probe of the SIET. We aimed to apply the SIET in the gills of adult zebrafish, detect branchial H^+^ activities, and test the hypothesis that branchial H^+^ is excreted through HA, and the H^+^-excreting capacity is enhanced under acidic water environments. This is the first study that successfully analyzed the ion-transporting functions in adult gills in vivo. We proved that zebrafish gills excrete H^+^ via apical HA (and also Nhe), and the H^+^-excreting capacity is enhanced under acidic situations by increasing the HR ionocyte number and the expression of HA in the gills. These findings suggest that SIET is competent for in vivo detection in fish gills, and represent a breakthrough in fish physiology study.

## 2. Results

### 2.1. Removal of the Opercula Did Not Contribute to High Mortality or Abnormal Oxygen Consumption

**Figure 1:**
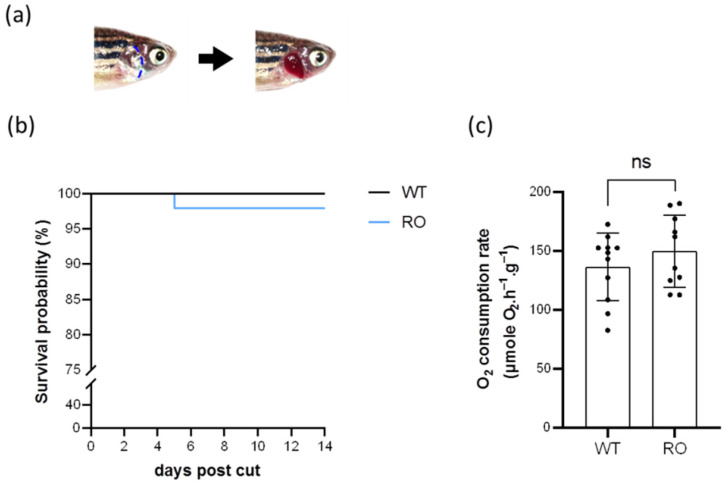
Effects of removal of the opercula on the survival rate and oxygen consumption rate. The opercula were cut along the blue dashed line, and the schema of opercula-removed zebrafish is shown (**a**). The survival rate (N = 50) (**b**) and oxygen consumption rate (N = 10–11) (**c**) were recorded after the opercula were removed. Values are the mean ± SD. Student’s *t*-test. ns = not significant. WT = wild type. RO = removal of the opercula.

To directly detect branchial H^+^ activities with the scanning ion-selective electrode technique (SIET), the opercula of adult zebrafish were cut and removed two weeks before acid acclimation or inhibitor treatments. The schema of fish without the opercula is shown in Figure 1a. The survival rate of the zebrafish without the opercula was >95% (Figure 1b). The oxygen consumption rate did not significantly change after removal of the opercula (Figure 1c).

### 2.2. H^+^ Activities Could Be Detected at Different Positions in the Gills

**Figure 2:**
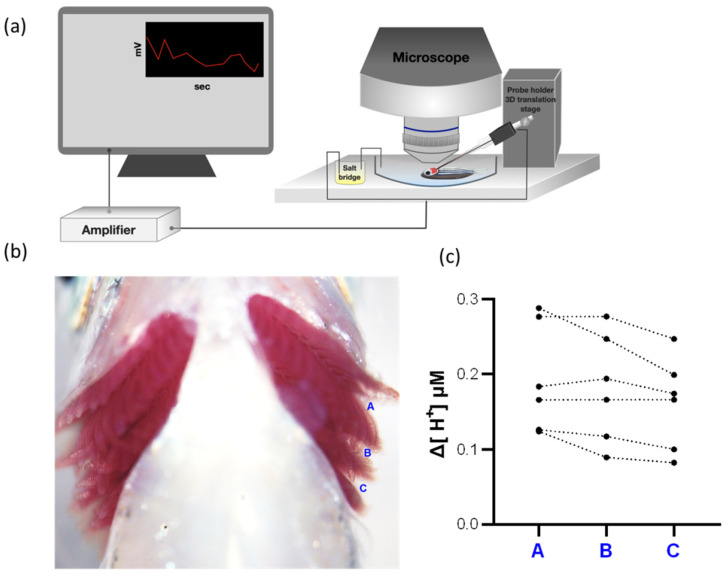
H^+^ activities at different positions in the gills. The diagram of the scanning ion-selective electrode technique (SIET) device is shown (**a**). Three measured positions (A, B, and C) between each gill arch are shown in (**b**). The H^+^ gradients at different positions of the gills were measured by the SIET (N = 6) (**c**). The dotted line represents the same fish.

Fish were placed on the recording stage of the SIET as presented in Figure 2a; H^+^ activities at the interspaces of the gills were measured with a H^+^-selective probe, including spaces A, B, and C (Figure 2b). The Δ[H^+^] of the three spots was determined by calculating the difference between the target spot and background. Similar Δ[H^+^] was detected in spaces A, B, and C in zebrafish (Figure 2c). Hence, we selected space B for H^+^ detections in the following SIET experiments.

### 2.3. Acidic Environments Increased the Number of HR Ionocytes

**Figure 3:**
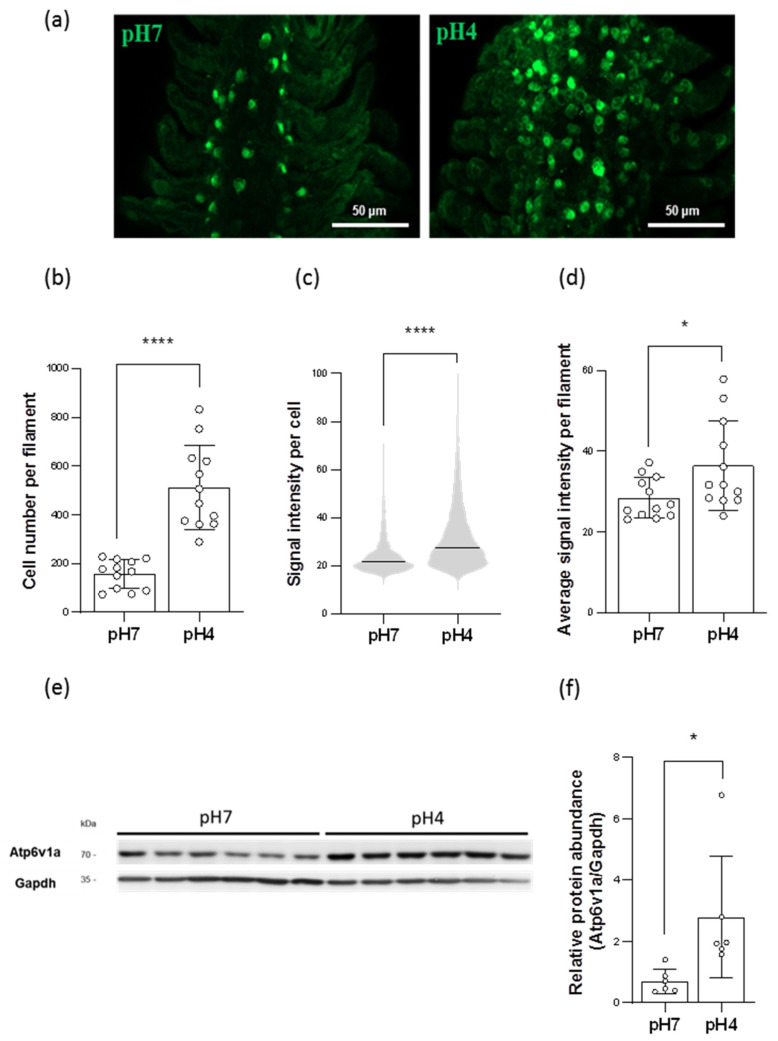
Effects of acid acclimation on the number of HR ionocytes and the expression of HA in the gills. The number of HR ionocytes and the fluorescence intensity of Atp6v1a signals in the gills were analyzed by immunofluorescence (IF) after acid acclimation (**a**). HR ionocyte number of filaments (N = 12) (**b**), mean fluorescence intensity of individual HR ionocytes (N = 1894–6144) (**c**), and average mean fluorescence intensity of HR ionocytes of filaments (N = 12) (**d**) were presented. The protein expression of HA in the gills acclimated to acid environments was analyzed by Western blot. The blots showed the bands with molecular weights corresponding to Atp6v1a and Gapdh (approximately 70 kDa and 35 kDa, respectively) (**e**). The protein expression of HA was quantified and normalized to Gapdh (N = 6) (**f**). Values are the mean ± SD or SEM. Student’s *t*-test or Mann–Whitney *U* test, * *p* < 0.05, **** *p* < 0.0001.

To investigate the H^+^-excreting mechanisms in the gills, the zebrafish were acclimated to acidic FW (pH 4) for 7 days. Immunofluorescence (IF) staining with the anti-Atv6v1a antibody showed that the number of HR ionocytes in the gills was increased when the fish were acclimated to acidic environments (Figure 3a,b). In addition, the results of the quantitative real-time polymerase chain reaction (qRT-PCR) revealed that the mRNA expression of *gcm2*, a transcription factor for the differentiation of HR ionocytes [22], was upregulated in acid-acclimated gills (Figure S1e).

### 2.4. Acidic Environments Increased the Expression of HA and Other H^+^ Excretion-Related Transporters/Enzymes

IF staining with anti-Atv6v1a antibody showed that the signal intensity of single HR ionocytes was increased in acid-acclimated gills (Figure 3c,d), suggesting that the protein expression of HA in HR ionocytes was upregulated. Consistent with this, the results of the qRT-PCR and Western blot also suggested that the expression of HA in acid-acclimated gills had increased (Figure 3e,f and Figure S1a); moreover, the mRNA expression of anion exchanger 1b (Ae1b) and carbonic anhydrase 2 (Ca2) was also stimulated (Figure S1c,d).

### 2.5. Acidic Environments Promote Plasma Membrane HA Accumulation

**Figure 4:**
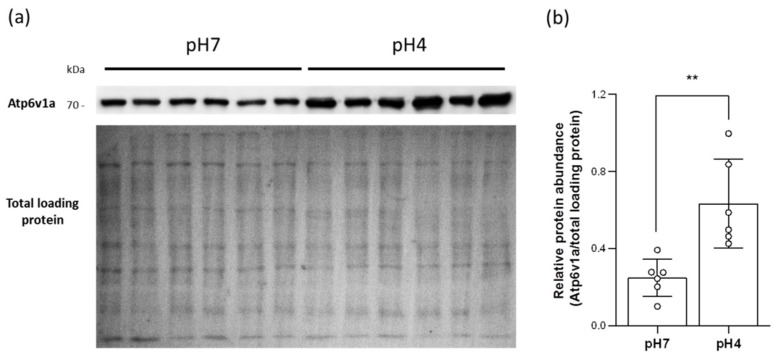
Effects of acid acclimation on the HA accumulation in the plasma membranes of the gills. The protein expression of HA in the branchial plasma membranes acclimated to acid environments was analyzed by Western blot. The blots showed the band with a molecular weight corresponding to Atp6v1a (approximately 70 kDa), and the PVDF membrane stained by ponceau S showed bands of total loading proteins (**a**). The protein expression of HA was quantified and normalized to total loading protein (N = 6) (**b**). Values are the mean ± SD. Student’s *t*-test, ** *p* < 0.01.

To confirm whether more HA proteins are accumulated in the plasma membranes under acidic situations, the branchial membrane protein was isolated to conduct Western blot. The results showed the HA expression in the plasma membranes was increased in acid-acclimated gills (Figure 4), suggesting that acidic FW not only triggers and enhances HA expression but also promotes plasma membrane HA accumulation.

### 2.6. Branchial H^+^-Excreting Capacity Was Elevated after Acid Acclimation

**Figure 5:**
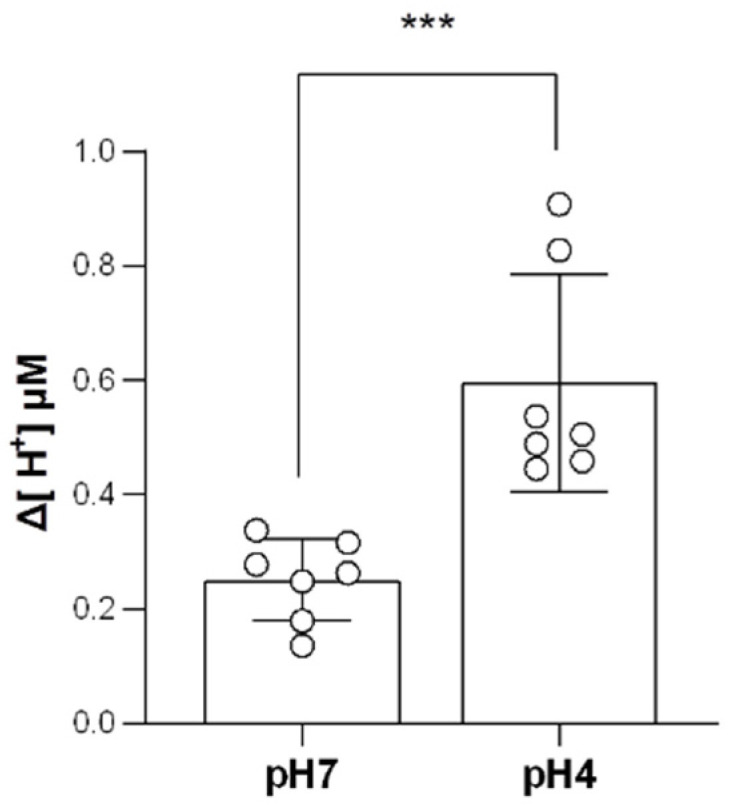
Effects of acid acclimation on the branchial H^+^-excreting capacity. The H^+^ gradient at the gills of adult fish was measured by the SIET after 7 days of acclimation to acidic conditions. Values are the mean ± SEM (N = 7). Mann–Whitney *U* test, *** *p* < 0.001.

Owing to the increasing HA expression and HR ionocyte number observed above, we next investigated whether the H^+^-excreting function was enhanced under acidic situations. Using the SIET, the calculated Δ[H^+^] of acid-acclimated gills was significantly higher than those of the control groups, suggesting that the branchial H^+^-excreting capacity was elevated (Figure 5).

### 2.7. HR Ionocytes Excreted H^+^ through HA and Nhe

**Figure 6:**
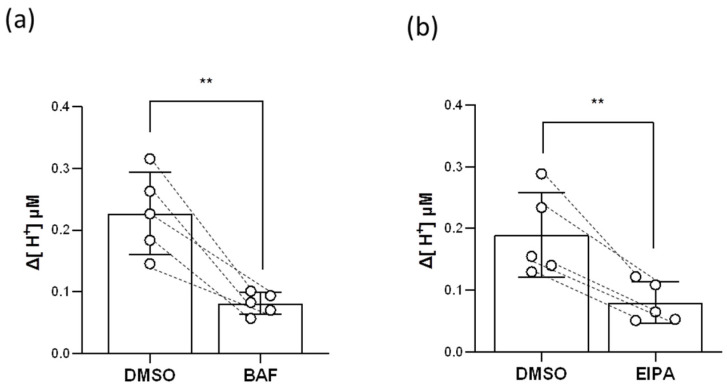
Effects of BAF and EIPA on the branchial H^+^-excreting capacity. H^+^ gradients at the gills were analyzed before and after BAF (**a**) or EIPA (**b**) treatments by the SIET. The dashed line represents the same fish. Values are the mean ± SD (N = 5). Student’s *t*-test, ** *p* < 0.01. BAF = bafilomycin A1. EIPA = 5-ethylisopropyl amiloride.

Both HA and Nhe3b are apically localized in the HR ionocytes of zebrafish gills and can transport H^+^ [8], so we examined the inhibitory effects of the HA inhibitor (bafilomycin A1, BAF, Selleck Chemicals LLC, Houston, TX, USA) and the Nhe inhibitor (5-ethylisopropyl amiloride, EIPA, Selleck Chemicals LLC, Houston, TX, USA) on branchial H^+^ transport functions. The results of the SIET showed that both BAF and EIPA impaired 50–60% of H^+^ activities in the gills (Figure 6), suggesting that branchial H^+^ is excreted via HA and Nhe.

## 3. Discussion

The present study successfully developed an in vivo detecting method for analyzing ion transport functions in adult fish gills. We demonstrated that zebrafish gills excrete H^+^ via HA and Nhe. This excretion function can be elevated by upregulating the expression of HA and increasing the number of HR ionocytes. Our breakthrough in gill functional analyses reveals the branchial acid excretion mechanisms in zebrafish and also provides a powerful platform for studying iono- and osmoregulation in adult fish gills.

The gills of teleosts possess a complex stereostructure and are protected by the opercula, which are not accessible to physiological detections with an electrode. Similar to the gill epithelium but flat, the opercular epithelium contains a high density of ionocytes [23,24,25,26]. Previous studies on branchial ion regulation used the opercular membranes as an alternative platform to analyze the ion transport functions of ionocytes. The opercular epithelium of seawater (SW)-acclimated teleosts was isolated and mounted on a Lucite/Ussing chamber to analyze short-circuit currents, transepithelial potential differences, and ion fluxes, allowing the researchers to explore the Na^+^- and Cl^−^-excreting functions of ionocytes [27,28,29]. In addition, the Na^+^/Cl^−^/Ca^2+^ uptake functions of opercular ionocytes were investigated in FW-acclimated killifish and tilapia [26,30,31]. However, no electrophysiological method has successfully identified the acid excretion functions of opercular or branchial ionocytes. Although BCECF-AM (a pH-sensitive dye) is a known pH indicator for monitoring intracellular pH [32], it cannot be directly used to illustrate whether the opercular or branchial ionocytes excrete acid in vivo. For the first time, the present study probed branchial H^+^ activities with a H^+^-selective electrode of the SIET (Figure 2, Figure 5, and Figure 6). Actually, the detected H^+^ may come from respiratory acid and transporter-based H^+^ excretion; thus, a HA inhibitor (BAF) and a Nhe inhibitor (EIPA) were used to prove that the partial H^+^ detected in the gills was indeed excreted through HA and Nhe (Figure 6). As such, our new application of the SIET in fish warrants further studies on adult gills to explore the ion uptake/excretion functions in FW or SW fish, other than those of branchial H^+^ excretion.

HR ionocytes are considered the main sites of acid–base regulation in zebrafish gills. Studies on embryonic zebrafish have demonstrated that H^+^ excretion via HA results in the generation of intracellular HCO_3_^−^ by cytosolic carbonic anhydrase 2 (Ca2) and the absorption of HCO_3_^−^ into the interstitium by basolateral Ae1b. Moreover, excreting H^+^ across the apical membranes facilitates the deprotonation of intracellular NH_4_^+^ and the excretion of NH_3_ by apical rhesus C glycoprotein 2 (Rhcg2). The excreted NH_3_ then combines with extracellular H^+^ extruded by HA (or Nhe3b) and is converted into NH_4_^+^. That is, HA promotes HCO_3_^−^ uptake as well as the NH_4_^+^ excretion functions of HR ionocytes [33]. Though both apical HA and Nhe3b excrete H^+^, most of the extruded H^+^ is believed to come from HA. Our previous works in embryonic zebrafish showed that the mRNA expression of Nhe3b was much lower than HA [34]; besides, HA inhibitor (BAF) treatments suppressed almost 70–75% of the H^+^ activities in the skin, and Nhe inhibitor (EIPA) treatments only decreased 15–20% [35]. However, we observed that EIPA impaired about 50–60% of the H^+^ activities in the gills (Figure 6b), meaning that Nhe3b contributes more to H^+^ excretion in adult gills than in embryonic skin. Indeed, branchial Nhe3b and HA showed similar mRNA expression levels under pH 7 FW conditions (Figure S1a,b). These data imply that HA and Nhe3b may have similar contributions to branchial H^+^ excretion when zebrafish live in normal FW. Of course, EIPA is not a specific inhibitor for Nhe3b, so we could not exclude the possibility that other Nhe isoforms expressed in the gills may contribute to H^+^ excretion.

Interestingly, zebrafish tend to utilize HA-based H^+^ excretion under acidified environments. Acid-acclimated zebrafish upregulated the expression of HA, Ae1b, and Ca2 in the gills (Figure 3c–f and Figure S1a,c,d), and promoted the HA accumulation in plasma membranes (Figure 4); in addition, increased expression of *gcm2*, a transcription factor that differentiates HR ionocytes [22], further enriched the number of HR ionocytes in acid-acclimated gills (Figure 3a,b and Figure S1e). These increased transporter expressions and ionocyte number enhanced branchial H^+^ excretion capacities (Figure 5). Together with the evidence that HA is apically expressed in branchial HR ionocytes [8], the enhanced H^+^ excretion capacities further implied that acidic FW triggered more HA being accumulated in the apical membranes of HR ionocytes. Notably, acidic environments downregulated the Nhe3b expression (Figure S1b) and decreased the Nhe3b-expressing cells in the gills [11], illustrating that HA-based H^+^ excretion machinery rather than Nhe3b appears to play a dominant role in acid–base regulation in zebrafish gills under acidic stress. Compared to the responses to pH 4 FW in embryos [12,16,34], the major principal of H^+^ excretion mechanisms is similar in zebrafish gills and embryonic skin acclimated to acidic environments.

In terms of transporter expression and functions, mammalian α-intercalated cells (α-ICs), abundant in the outer medullary collecting duct (OMCD), are analogous to zebrafish HR ionocytes. α-ICs actively extrude H^+^ into the lumen through apical HA and H^+^/K^+^-ATPase, which produces urine with higher acidities and is critical for renal acid excretion [36]. It has been well-reported that metabolic or respiratory acidosis causes the remodeling of the collecting duct, including an increased number and a morphological change in α-ICs. Increased surface areas allow more HA and AE1 to be inserted into the apical and basolateral membranes of α-ICs, respectively, in acidotic rodents [36,37,38]. Microperfusion experiments functionally revealed that acidosis stimulates H^+^ excretion in OMCD [39,40]. However, it is still difficult or impractical to conduct in vivo functional assays in the kidney of a living animal. As with the ionoregulatory functions of a mammalian kidney, it is much easier to analyze the ion-transporting functions of fish gills in vivo when they are directly exposed to the environment. Using our novel method in the present study, fish gills can serve as a surrogate system to explore renal physiology.

In summary, the SIET is competent for analyzing ion transport functions in fish gills. We applied this technique in zebrafish gills and identified branchial acid-excreting mechanisms. Zebrafish mainly utilize HA-based H^+^ excretion machinery by controlling the expression of H^+^ excretion genes and the number of HR ionocytes to deal with the acidified FW environments. In addition to H^+^-excreting functions, this technique could be used to analyze the transport functions of other ions (e.g., Na^+^, K^+^, Cl^−^, Ca^2+^, and NH_4_^+^) with different ion-specific probes. The present study not only enhances our understanding of branchial ion regulation but paves a new path for the study of fish iono- and osmoregulation mechanisms.

## 4. Materials and Methods

### 4.1. Experimental Animals

Mature zebrafish (*Danio rerio*) were reared in circulating local tap FW at 28 °C under a 14:10 h light/dark photoperiod at the Institute of Cellular and Organismic Biology (ICOB), Academia Sinica, Nangang, Taipei, Taiwan. The experimental protocols were approved by the Academia Sinica Institutional Animal Care and Utilization Committee (approval no.: BSF0415-00003197), and all methods were performed in accordance with relevant guidelines and regulations. Fish were first anesthetized in FW containing 0.3 mg/L ethyl 3-aminobenzoate (Sigma-Aldrich, Taipei City, Taiwan) before we removed the opercula or sacrificed them. To detect the branchial H^+^ activities by the SIET, the opercula were cut and removed 2 weeks before the acclimation/pharmacological experiments. The survival rates of the fish were recorded for 2 weeks after removal of the opercula, and then zebrafish were starved for one week before measuring the oxygen consumption rate.

### 4.2. Measurements of the Oxygen Consumption Rate

Fish were transferred to the glass respiration chamber filled with FW individually. To detect the changes in oxygen concentration of the FW, a fiber-optic oxygen sensor (Oxy-4 mini) (PreSens, Regensburg, Germany) was equipped to the chamber and connected to a multi-channel oxygen meter (Oxy-4 mini) (PreSens, Regensburg, Germany). After calibration with oxygen-saturated FW and FW with no oxygen (10% sodium bisulphate solution), the oxygen concentration of the FW was monitored every 5 s for 1 h. Oxygen concentration decreased linearly over time. This decrease was used to calculate the oxygen consumption rate of zebrafish. One chamber without fish was used to analyze background respiration from microorganisms.

### 4.3. Acid Acclimation Experiment

The acidic FW (pH 4) was prepared by adding H_2_SO_4_ to local tap aerated FW. Adult zebrafish were acclimated to acidic FW for 7 days without feeding. To maintain pH at a stable value, the prepared acidic FW stock was continuously pumped into the experimental tank with an electrical pump. The pH values of all experimental media were checked using a pH meter (Conventional pocket meters ProfiLine pH 3310) (WTW, Weilheim, Germany). No mortality was observed during the experiments.

### 4.4. Complementary DNA (cDNA) Preparation

Adult gills isolated from one individual were pooled into one sample. Samples were homogenized in TRIzol Reagent (Invitrogen, Waltham, MA, USA). Following the manufacturer’s protocol, total RNA was extracted and purified. DNase I (Roche, Basel, Switzerland) was then used to remove genomic DNA. The quality and quantity of the total RNA were checked using NanoDrop 2000 (Thermo Scientific, Waltham, MA, USA). Total RNA (2 μg) was used to synthesize cDNA with SuperScript IV reverse transcriptase (Thermo Scientific, Waltham, MA, USA), following the manufacturer’s protocol.

### 4.5. Quantitative Real-Time Polymerase Chain Reaction (qRT-PCR)

We used a Light Cycler real-time PCR system (Roche, Basel, Switzerland) to conduct qRT-PCR following a previous protocol [41]. The expression of ribosomal protein L13a (*rpl13a*) was used as an internal control for zebrafish. The primer sets used for qRT-PCR are provided in Supplementary Table S1. The amplification efficiency was confirmed by serial cDNA dilution for each primer set, and the specificity of primer sets was checked by Sanger sequencing of the amplicons.

### 4.6. Immunofluorescence (IF)

The gills of adult zebrafish were collected and fixed from three individuals, and whole mount IF was then performed following a previous study [11]. A rabbit Atp6v1a polyclonal antibody (Proteintech, Rosemont, IL, USA) (1:100 dilution) and an Alexa Fluor 488 goat anti-rabbit immunoglobulin G (IgG) (Invitrogen, Waltham, MA, USA) (1:200 dilution) were used. The middle parts of the filaments within second pairs of the adult gills [11] were selected for imaging. For each individual, Atp6v1a-positive cells (HR ionocytes) within a given length (400 μm in the distal edge of the filament) from four randomly selected filaments were counted and fluorescence intensity of the cells quantified. Sample images were obtained with LSM 980 (Zeiss, Jena, Germany). Imaris 9.8.2 (Oxford Instruments plc, Abingdon, UK) was then used to analyze the number and fluorescence intensity of Atp6v1a-positive cells (HR ionocytes).

### 4.7. Western Blot

For total protein extraction, adult gills isolated from one individual were pooled into one sample, homogenized in ice-cold SEID buffer (150 mM sucrose, 10 mM EDTA, 50 mM imidazole, 0.1% sodium deoxycholate, pH 7.4) with cOmplete Protease Inhibitor Cocktail (Roche, Basel, Switzerland), and centrifuged at 13,000× *g* for 20 min at 4 °C. The supernatant (total protein) was collected. For membrane protein extraction, adult gills collected from three individuals were pooled into one sample and homogenized in ice-cold SEI buffer (150 mM sucrose, 10 mM EDTA, 50 mM imidazole, pH 7.4) with cOmplete Protease Inhibitor Cocktail (Roche, Basel, Switzerland). According to Tang and Lee (2011) [42], the homogenate was centrifuged at 13,000× *g* for 10 min at 4 °C. The supernatant was then collected and centrifuged at 20,800× *g* for 1 h at 4 °C. After the centrifugation, the supernatant (cytosolic fraction) was collected, and the pellet (membrane fraction) was dissolved with ice-cold SEID buffer. A mixture of the homogenate (30 μg total protein or 15 μg membrane protein) and 6X Protein Sample Dye (β-Me) (ACE Biolabs, Taoyuan City, Taiwan) was first denatured at 95 °C for 5 min and then followed a previous protocol [43]. Atp6v1a Polyclonal antibody (Proteintech, Rosemont, IL, USA) (1:1000 dilution), Gapdh antibody (GeneTex, Hsinchu City, Taiwan) (1:5000 dilution), Na^+^/K^+^-ATPase α Antibody (H-300) (Santa Cruz, Dallas, Texas, USA) (1:1000 dilution), and Goat anti-Rabbit IgG (H+L) Secondary Antibody, HRP (Invitrogen, Waltham, MA, USA) (1:5000 dilution) were used for immunoblotting. Signals were obtained by adding WesternBright ECL (Advansta, San Jose, CA, USA). The amounts of the loaded membrane protein were estimated by Ponceau S Staining Solution (Thermo Scientific, Waltham, MA, USA). Images were acquired by UVP BioSpectrum 600 and quantitated by Fiji [44]. The isolation quality test of cytosolic and membrane fractions is shown in Supplementary Figure S2.

### 4.8. Scanning Ion-Selective Electrode Technique (SIET)

The SIET was performed at room temperature (26–28 °C) in an agar chamber filled with FW-recording medium (0.2 mM NaCl, 0.05 mM KH_2_PO_4_, 0.05 mM K_2_HPO_4_, 0.2 mM CaSO_4_, 0.2 mM MgSO_4_, 300 µM MOPS buffer (Sigma-Aldrich, Taipei City, Taiwan), 0.3 mg/L ethyl 3-aminobenzoate (Sigma-Aldrich, Taipei City, Taiwan), pH 7.0). Anesthetized fish were laid out in the agar chamber with the belly facing upward (Figure 2a). To measure the H^+^ gradients (represented as Δ[H^+^]) between the target spot and background, a H^+^-selective microelectrode was prepared to record the H^+^ activities with ASET software, following the previous study [16]. The background H^+^ activities were first recorded before the fish were placed in the chamber. Once the fish were set on the chamber, the H^+^ activities at three target spots between each pair of gills (the middle part of the gills) were then detected (Figure 2b). Branchial Δ[H^+^] was then calculated.

### 4.9. Pharmacological Treatments

The stocks of 8 mM bafilomycin A1 (BAF) (Sigma-Aldrich, Taipei City, Taiwan) and 400 mM 5-ethylisopropyl amiloride (EIPA) (Sigma-Aldrich, Taipei City, Taiwan) were prepared in dimethyl sulfoxide (DMSO). The original H^+^ activities of zebrafish gills were recorded, and then the zebrafish were subjected to inhibitor treatments to analyze the inhibitory effects on branchial H^+^ excretion. For the BAF experiments, zebrafish were incubated in FW-recording medium containing 10 μM BAF for 3 min when detecting H^+^ activities with the SIET. For the EIPA experiments, zebrafish were pre-incubated in FW containing 100 μM EIPA for 1 h and then transferred to FW-recording medium containing 100 μM EIPA for SIET recording. The concentrations of BAF and EIPA were selected based on previous studies [9,45].

### 4.10. Statistical Analysis

Values are presented as the mean ± standard deviation (SD) for the parametric data and the mean ± standard error of the mean (SEM) for nonparametric data. The Shapiro–Wilk normality test was first applied to evaluate the normality of the datasets before statistical analysis was performed. Data from each group were analyzed using a Student’s *t*-test or Mann–Whitney *U* test when applicable. Statistical analysis was performed using Prism 8.4.2. (GraphPad, CA, USA).

## Data Availability

The raw data used for all statistical analyses can be found at https://drive.google.com/drive/folders/17J5hO9Yw8Svr-MKb8DB78AUwN9o_BsWW?usp=sharing (accessed on 27 March 2022).

## Supplementary Materials

The following are available online at https://www.mdpi.com/article/10.3390/ijms23084419/s1.

## Abbreviations

Ae1b	Anion exchanger 1b
BAF	Bafilomycin A1
Ca2	Carbonic anhydrase 2
EIPA	5-ethylisopropyl amiloride
FW	Freshwater
HA	H^+^-ATPase
HR	H^+^-ATPase-rich
IF	Immunofluorescence
Nhe	Na^+^/H^+^ exchanger
Rhcg2	Rhesus C glycoprotein 2
RO	Removal of the opercula
SIET	Scanning ion-selective electrode technique
SW	Seawater
