# Supplementary material for: In Vivo Functional Assay in Fish Gills: Exploring Branchial Acid-Excreting Mechanisms in Zebrafish
Source: Int J Mol Sci. 2022 Apr 16;23(8):4419. doi: 10.3390/ijms23084419 (PMC9031880; doi:10.3390/ijms23084419)
Supplement: Supplementary file 1 [file ijms-23-04419-s001.zip › Figure S1.pdf]

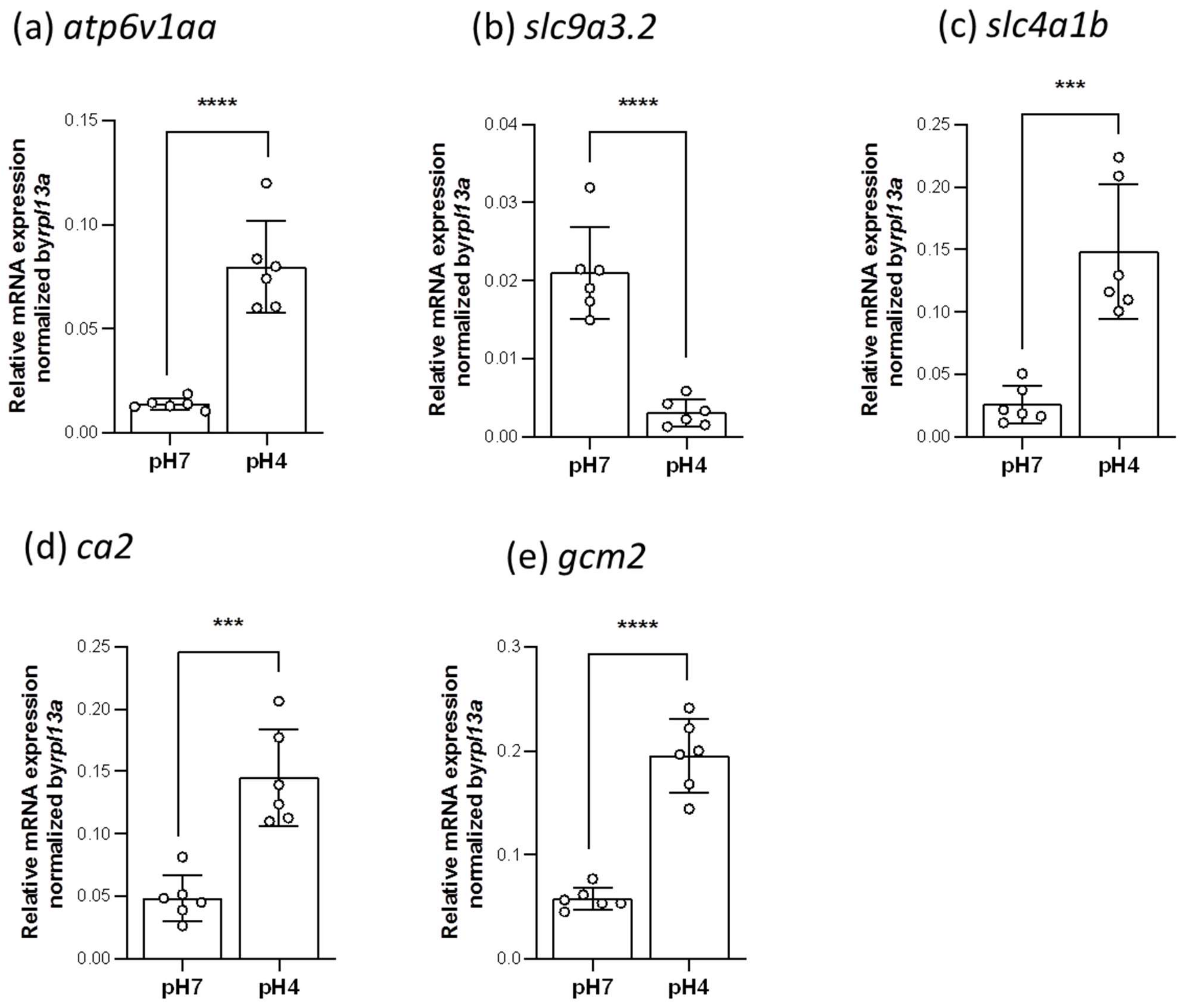

Figure S1. Effects of acid acclimation on the mRNA expression of acid excretion-related genes in the gills. The mRNA expression levels of *atp6v1aa* (HA) (a), *slc9a3.2* (Nhe3b) (b), *slc4a1b* (Ae1b) (c), *ca2* (d), and *gcm2* (e) in adult gills were analyzed by quantitative real-time polymerase chain reaction (qRT-PCR) after acid acclimation. Data were normalized to *rpl13a*. Values are the mean ± SD (N=6). Student's *t*-test, ***$p < 0.001$, ****$p < 0.0001$.
