# Supplementary material for: In Vivo Functional Assay in Fish Gills: Exploring Branchial Acid-Excreting Mechanisms in Zebrafish
Source: Int J Mol Sci. 2022 Apr 16;23(8):4419. doi: 10.3390/ijms23084419 (PMC9031880; doi:10.3390/ijms23084419)
Supplement: Supplementary file 1 [file ijms-23-04419-s001.zip › Figure S2.pdf]

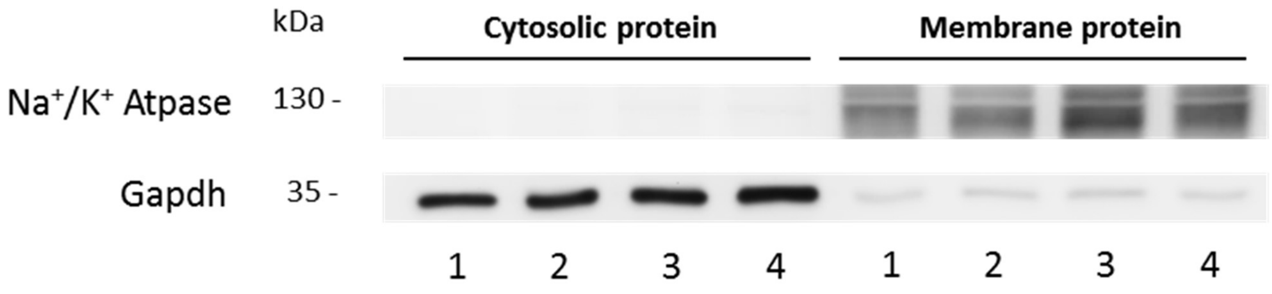

Figure S2. Isolation quality test of the cytosolic and membrane fractions. The cytosolic and membrane proteins were extracted from the gills of wild-type zebrafish. 15 µg cytosolic and membrane proteins were loaded for western blot (N=4). The blots showed the bands with molecular weights corresponding to Na^+/K^+ Atpase and Gapdh (approximately 130 kDa and 35 kDa, respectively). The same number represents the proteins isolated from the same sample.
