# Supplementary material for: In Vivo Functional Assay in Fish Gills: Exploring Branchial Acid-Excreting Mechanisms in Zebrafish
Source: Int J Mol Sci. 2022 Apr 16;23(8):4419. doi: 10.3390/ijms23084419 (PMC9031880; doi:10.3390/ijms23084419)
Supplement: Supplementary file 1 [file ijms-23-04419-s001.zip › Table S1.pdf]

Table S1. Primer sets used for qRT-PCR.

Name	Ensembl ID		Sequence (5' to 3')
rpl13a	ENSDARG00000116541	Forward	CCTCGGTCGTCTTTCCGCTATTG
		Reverse	CAGCCTGACCCCTCTTGGTTTTG
atp6v1aa	ENSDARG00000113437	Forward	GAGGAACCACTGCCATTCCA
		Reverse	CAACCCACATAAATGATGACATC
slc9a3.2	ENSDARG00000036722	Forward	TGCAGACAGCGCCTCTAGC
		Reverse	TGTGGCCTGTCTCTGTTTGC
slc4a1b	ENSDARG00000024560	Forward	GGGAATCACATCACTGAGTGGT
		Reverse	GCATCTTACTGGTGCTGACTC
ca2	ENSDARG00000014488	Forward	TCGTCTGCAAACAGTCCATCA
		Reverse	TCCGAGAGGCCCTTCATTT
gcm2	ENSDARG00000045413	Forward	TCCCTGTGGTTATGACTTTGCA
		Reverse	TGGACTTGAGCCATGAGACACT
